# Research on Risk Contagion among Financial Submarkets in China Based on Complex Networks

**DOI:** 10.3390/e24081120

**Published:** 2022-08-14

**Authors:** Yuhua Xu, Yue Zhao, Mengna Liu, Chengrong Xie

**Affiliations:** 1 School of Finance, Nanjing Audit University, Nanjing 211815, China; 2 School of Statistics and Mathematics, Nanjing Audit University, Nanjing 211815, China

**Keywords:** risk contagion, financial sub-markets, complex network

## Abstract

As the COVID-19 outbreak has an impact on the global economy, there will be interest in how China’s financial markets function during the outbreak. To investigate the path of risk contagion in China’s financial sub-markets before and after the COVID-19 outbreak, we divided the 2016–2021 period into two phases. Based on the time of the COVID-19 outbreak, we divided the new stage of economic development into pre-epidemic and post-epidemic stages and employed the DCC-GARCH model to investigate the dynamic correlation coefficients among the financial sub-markets in China. Furthermore, we employed complex network theory and the minimum tree model to describe the risk contagion path between two-stage Chinese financial submarkets. Finally, we provided pertinent recommendations for investors and policymakers and conducted a brief discussion based on the findings of the research.

## 1. Introduction

Since the 1990s, the links among the global economy, trade, and finance have been significantly strengthened, as have the effects of financial cross-market risks. When one sub-market of the financial market experiences a shock, the other sub-market will also fluctuate as a result of the risk conduction effect. In extreme cases, systemic financial risks could even erupt. Both the 2008 financial crisis and the 2015 stock market crash had led to the spread of systemic risks in China’s financial markets, the Chinese government has put forward new goals and requirements for economic development, known as the “New Normal”. However, the COVID-19 epidemic in 2019 has not only affected the normal life of the Chinese people but also the development of the production and investment of Chinese enterprises. Against the background of the epidemic, China’s financial market has entered a unique “New Normal”. In the context of the “New Normal” of China’s financial market, many scholars have studied some characteristics of China’s financial markets and financial institutions by using various techniques and methods and obtained some meaningful conclusions. For example, Ribeiro GT considered that the prediction of stock price volatility was important for financial companies and investors to measure and manage market risk and support financial decision-making [1]. Therefore, this paper is meant to study the impact of COVID-19 on financial enterprises in China from 2016 to 2021, hoping to provide some suggestions for the stable growth of such markets.

With the development of network science, more and more researchers used the properties of complex networks to study the contagion risk of financial markets. For example, the variant of artificial neural network has been proposed, such as Echo state network (ESN), which based on the reservoir computing paradigm, have been proposed to improve time series prediction. Yang conducted an empirical study about U.S. Treasury bonds, global stock indices and commodities by identifying volatility spillover networks and found that the U.S. stock market was the center of the international volatility spillover network, and its volatility spillover effect has intensified since 2008 [2]. Zhang used complex network theory and GARCH-BEKK model to study the causes of financial risks and the spatial spillover effects of G20 stock market volatility [3], the dynamic volatility spill-over effect between stock indexes and commodity futures markets was empirically examined by Sang using the theory. It was determined that the index and commodity futures had a positive association [4]. Xie explained the evolution of extreme risk spillovers in China’s financial sub-market by using directed weighted complex networks. The empirical results show that although the energy secondary market continues to evolve over the five stages, it remains the main recipient of significant upside risk spillovers. After the financial crisis, there were a strong extreme risk spillover effect between the commodity and bond submarkets due to their increased ability to transmit extreme risk spillovers in the network [5]. Hao constructed a risk contagion network in China’s financial markets and studied the changes of risk spillovers in the financial system in the five years before and after the outbreak of COVID-19 [6]. Based on the idiosyncratic volatility spillover effects among the money market, capital market, commodity trading market, foreign exchange market, real estate market, and gold market, Gong constructed a complex information spillover network and investigated the characteristics of risk contagion within the financial system from the network perspective [7]. In order to determine the risk center and development process of the crisis, Liu employed the spillover index and complex network methods to quantify the intensity and direction of risk spillovers in China’s financial market from a static and dynamic perspective [8]. Based on historical data from China from January 1996 to December 2016, Zhao established a volatility spillover model of financial markets and demonstrated the cross-market volatility spillover impact [9]. Based on the GARCH-BEKK model, Li built a volatility spillover network connecting several stock market locations in China and examined the risk contagion effect between these regions [10]. Based on a regime switching skew-normal (RSSN) model, Luo investigated the linear and non-linear dependence structures of risk contagions between global crude oil futures markets and China’s agricultural futures markets, identifying the contagion channels under both stable and erratic oil market conditions [11]. According to the authors’ findings by Feng, cross-shareholding networks were said to strengthen stock market linkages and led to risk contagion. Based on his research, cross ownership can reduce the impact of minor and sustained external shocks and volatility is highly sensitive to changes in network density [12].

Furthermore, some scholars have studied the characteristics of financial institutions based on complex networks. For example, Huang first studied the return spillover effect among financial institutions. Then, the Granger causality in vector autoregressive (VAR) model is used to construct a core revenue network. Finally, he calculated the impact of the network centrality of financial institutions on measuring the scientific importance of financial institutions [13]. Chuang investigated the potential effects of interconnectivity inside a securities firm on stock returns and suggested a financial network model to mimic the information transfer process of investors within a securities company [14]. Ouyang used the directed network method to measure the systemic risk contagion effect of China’s banking industry, and the results showed that with the outbreak of the crisis, the correlation degree of the financial system was continuously enhanced, and the overall connectivity of the network was continuously enhanced until it reached the maximum level. However, systemic risk tends to lag behind the overall connectivity of the network [15]. Utilizing data from 111 Chinese banks from 2013 to 2016, Chen concluded that the risk contagion faced by Chinese banks varied depending on the bank network structure and that raising capital levels helped banks be more resilient to credit and liquidity shocks [16]. Fan used empirical Bayesian techniques to integrate the correlation between institutional stock returns, sentiment and marginal expectation gap, and concluded that excessive links among financial institutions would produce contagion effects and make financial shocks more severe [17]. To the best of our knowledge, up to now, the introduction of DCC-GARCH model in complex networks to study risk contagion in financial markets has received little attention. Motivated by the above discussion, the main purpose of this paper is to discuss the changes of Chinese financial market before and after the COVID-19 from the perspective of risk contagion. In order to explore the dynamic correlation coefficients between China’s financial sub-markets, we first use the DCC-GARCH model. Next, we build complex networks, and the minimal spanning tree model is used to show the risk contagion path among Chinese financial sub-markets.

## 2. Theoretical Model Analysis

The theory of the DCC-GARCH model, complex network model, and minimum spanning tree model is introduced in this section. These models serve as theoretical underpinnings for the empirical study in the following chapter. In this study, DCC-GARCH model is used to explore the dynamic correlation coefficients among multiple markets. Based on the multi-agent and complex nature of financial sub-markets, a complex network model is proposed to capture the relationship between these markets and the route of infection.

### 2.1. Introduction to DCC-GARCH Model

The DCC-GARCH model is typically used to observe the volatility correlation among numerous financial institutions and estimate its dynamic correlation coefficient, in contrast to the GARCH model, which examines the conditional volatility of a single market.

#### 2.1.1. ARCH Model

**Definition** **1** ([18]). *The formula for establishing the ARCH model is as follows:*

(1) $$y_{t}={\lambda x}_{t}+\mu_{t},\mu_{t}/\varphi_{t}∼N\left(0,1\right),$$

(2) $$\delta^{2}=c_{0}+\sum_{i\;=1}^{p}\alpha_{i}\mu_{t\;-\;i}^{2}$$

Among them, p is the lag order, $y_{t}$ is the dependent variable, $x_{t}$ is the independent variable, $\mu_{t}$ is the random disturbance term, and $c_{0}$ is the constant term. $\alpha_{i}>0$ and $\sum_{i\;=1}^{p}\alpha_{i}>1$.

#### 2.1.2. GARCH Model

The characteristics of a sizable body of financial data have demonstrated that there are numerous nonlinear correlations in the financial market, which motivates people to build nonlinear models for the analysis of financial risk issues. Only a few nonlinear models are appropriate for financial sequence fitting despite the fact that there are several types of nonlinear models used in other fields. The GARCH model, which simulates and forecasts volatility, is a nonlinear financial model that is frequently used in research. The return series of financial assets’ volatility aggregation characteristics can be well described by the GARCH model. The volatility aggregation of generic financial series often implies that asset values will continue to experience modest variations after small fluctuations while also experiencing huge fluctuations after significant changes. The conditional variance of the residual term is modelled as a function of the squared residuals from earlier periods to produce the GARCH model.

**Definition** **2** ([19,20]). *The formula for establishing the GARCH(p,q) model is as follows:*

(3) $$\sigma_{t}^{2}=\alpha_{0}+\alpha_{1}\varepsilon_{t\;-1}^{2}+\ldots +\alpha_{p}\varepsilon_{t\;-\;p}^{2}+\beta_{1}\sigma_{t\;-1}^{2}+\ldots +\beta_{p}\sigma_{t\;-\;p}^{2}$$

Among them, p=1, q=1, the parameters used in financial data are more common.

#### 2.1.3. DCC-GARCH Model

To examine the fluctuation correlation among many series, the DCC-GARCH model is used. The conditional correlation dynamic correlation coefficient, which represents the strength of the correlation among the variables, is frequently calculated by using this model.

**Definition** **3** ([21]). *The DCC-GARCH model expression is as follows:*

(4) $$r_{t}=\mu_{t}+\varepsilon_{t},$$

$r_{t}$ is the column vector of returns; $\mu_{t}$ is the constant intercept term; $\varepsilon_{t}$ is the column vector of residuals. $\mu_{t}=E\left(r_{t}{|\varphi }_{t\;-1}\right)=E_{t\;-1}\left(r_{t}\right)$, $\varphi_{t}$ is the data set before period t.

$H_{t}$ is the time-varying conditional covariance matrix, expressed as

(5) $$H_{t}=\left[\begin{array}{ccc} h_{11,t} & \cdots & h_{1k,t}\\ \vdots & \ddots & \vdots \\ h_{k1,t} & \cdots & h_{\mathrm{kk},t} \end{array}\right]=D_{t}R_{t}D_{t}.$$

Among them, $D_{t}=\mathrm{diag}\left(\sigma_{i,t},\ldots ,\sigma_{n,t}\right)$ represents the diagonal matrix of conditional standard deviation elements, $R_{t}={\mathrm{diagQ}}_{t}^{-1/2}Q_{t}{\mathrm{diagQ}}_{t}^{-1/2}$ represents the dynamic correlation coefficient matrix, $Q_{t}=\left(1-\overset{M}{\underset{m=1}{\sum }}\alpha_{m}-\overset{N}{\underset{n=1}{\sum }}\beta_{n}\right)\;Q\;¯+\overset{M}{\underset{m=1}{\sum }}\alpha_{m}\left(\varepsilon_{t}-{\;m\varepsilon }_{t}^{*}-\;m\right)+\overset{N}{\underset{n=1}{\sum }}\beta_{n}Q_{t\;-\;n}$, the residual α represents the impact of the current information on the volatility of the next period, and the conditional variance β represents the persistence of yield volatility. If the coefficient, α+β<1, it means that the model is stable and there is an effective dynamic correlation. The closer the sum of α+β is to 1, the longer the fluctuation can last.

### 2.2. Application of GARCH Family Model

The stock market risk contagion process is examined using the GARCH model. These are the typical models: APARCH is a GARCH derivative model that takes into account leverage effect; TGARCH is known as Threshold Arch model, which illustrates that the influence of good news and bad news on conditional variance is different. The Absolute Arch Model, or ABSGARCH, lessens the magnitude of the intercept component by substituting the absolute value of the intercept term for the square of the intercept term. IGARCH, also known as the infinite variance GARCH model, simplifies the calculation by combining the two GARCH parameters into one parameter; The reason GARCH-M is sometimes known as the mean GARCH model is because it adds a variance variable to the mean equation. In the multivariate GARCH model, CCC, BEKK, and DCC are included. Although it has significant drawbacks, the conventional functional GARCH model can be utilized to characterize the heteroscedasticity of functional financial time series. For instance, it is unable to explain whether current information continues to have an effect on future conditional variance since it ignores the asymmetry of volatility. The dynamic variation of the conditional volatility may be constrained by the parameter’s non-negative constraint. The DCC-GARCH model illustrates the degree of influence and persistence of volatility correlation among various markets, whilst the BEKK-GARH model addresses the impact of its asymmetry orientation.

### 2.3. Complex Network Theory 

#### 2.3.1. Degree and Degree Distribution

Degree [21]: The mean of the degrees of all nodes ${\;V}_{i}$ in the network is called the average degree $k_{i}$ of the network, denoted as k.

(6) $$k=\frac{1}{N}\sum_{1}^{N}k_{i}.$$

N represents the number of nodes in this paper.

The degree of a node is defined as the number of edges connected to that node.

#### 2.3.2. Degree and Degree Distribution

The distance $d_{\mathrm{ij}}$ between two nodes $v_{i}$ and $v_{j}$ represents the number of shortest path edges among the nodes, and the diameter of the network refers to the maximum distance between two random nodes in the network, denoted as D. [21]

(7) $$D=\underset{1\leq i<j\leq N}{\max}d_{\mathrm{ij}}.$$

The average path length L of the network represents the average distance between two random nodes.

(8) $$L=\frac{1}{c_{N}^{2}}\sum_{1\leq i<j\leq N}d_{\mathrm{ij}}.$$

#### 2.3.3. Clustering Coefficient

The clustering coefficient $c_{i}$ of node $v_{i}$ refers to the ratio between the actual number of edges $E_{i}$ between the $k_{i}$ neighbor nodes of node $v_{i}$ and the total possible number of edges $c_{k_{i}}^{2}$, that is, $c_{i}=\frac{e_{i}}{c_{k_{i}}^{2}}$ [21]. The clustering coefficient C is the average of the clustering coefficients $c_{i}$ of all nodes $v_{i}$, namely

(9) $$C=\frac{1}{N}\sum_{i\;=1}^{N}c_{i},$$

### 2.4. Minimum Spanning Tree Model

#### 2.4.1. Krustal Algorithm

According to Kruskal’s algorithm, a forest is always the intermediate result. Every time an edge with the minimum weight and no cycles is chosen to join set A for the first set of n points, the two forests are combined. To assist in the identification of rings and edges, Disjoint Set Union is necessary.

Operation of the Kruskal edge: When making decisions, greedy thinking emphasizes making the best decision each time and making sure that a loop does not occur.

Its particular action: 

1. Initialization: Each vertex transforms into a forest on its own; 2. Add edges: To connect the two forests, choose the edge with the smallest weight out of all the edges. It is essential to guarantee that the chosen edge merged forest won’t produce a ring.

#### 2.4.2. Minimal Tree Diagram Model

The minimal tree graph model is an extension of the minimal spanning tree model, which describes a dendrogram that is produced from a unique root point in a directed graph, starting at the root point and going to all nodes in the graph.

**Definition** **4** ([10]). *Let be a directed graph if it has the following properties:* $G=\left(V,E\right)$

1. G does not contain directed rings;
2. There is a node $v_{i}$, which is not the end point of any arc, and other nodes in *V* are the end points of a unique arc, then G is a tree graph with $v_{i}$ as the root.

### 2.5. Basic Algorithm

The Zhu-Liu algorithm [22], which Zhu Yongjin and Liu Zhenhong devised in 1965, is the algorithm utilized in this study. Below, $v_{0}\;$ is the vertex identified as the root. The concepts of the minimum tree diagram and the minimum spanning tree are conceptually equivalent and both are based on point reduction and greed. A shrinking point is one where several points are treated as one, all edges connecting to it are treated as connecting to it, and all edges originating from it are treated as originating from the departure of the shrinking point.

1. From all arcs with $v_{i}\left(i\neq 0\right)$ as the end point, take the shortest one, and if $v_{i}$ is not the root node for a certain point and there is no incoming edge, the minimum tree graph cannot be generated, and the algorithm ends; if the root is removed If all nodes other than nodes have incoming edges, a subgraph $G^{\prime }$ with the smallest weight can be obtained, and the arc set in the subgraph $G^{\prime }$ is the shortest arc set;
2. After completing step 1, to obtain the shortest arc set, it is necessary to check whether there are directed cycles and contraction points in the connected graph. There are three cases: if there is no directed loop and no shrinkage points, the calculation is over, and the smallest tree graph with $v_{0}$ as the root is generated in step 1; if there is no directed loop but there are shrinkage points, it is necessary to expand the contraction point; if there is a directed loop, the directed loop needs to be contracted into a point to generate a new graph $G_{1}$;
3. The directed loop in the original graph G is contracted into a point. At this time, the edges belonging to the directed loop in the original graph *G* are contracted, while other arcs remain, thus obtaining a new graph $G_{1}$. Compared with the original graph G, in the new graph $G_{1}$, the direction of the length change of the arc with the contraction point as the end point remains unchanged, and the nature of whether to generate the minimum tree graph remains unchanged. At the same time, the operation of step 1 needs to be performed on the new graph $G_{1}$ until there is no directed cycle in the graph.
4. If the original graph G has a directed cycle, and the minimum tree graph $T_{1}$ of the new graph $G_{1}$ has been obtained, then all arcs in $T_{1}$ also belong to T. We can expand a contraction point of graph $G_{1}$ into a directed ring and remove arcs with the same end point in $T_{1}$, so that all other arcs belong to T.

## 3. Empirical Analysis

In this section, the DCC-GARCH model is used to obtain the dynamic correlation coefficient among financial submarkets, and then the minimum spanning tree method is used to intuitively explore the risk transmission among financial submarkets by building a complex network model.

### 3.1. Analysis of Sample Data of China’s Financial Market

The financial market is divided into six financial sub-markets in this paper; that is, in Table 1, the capital market, currency market, foreign exchange market, commodity market, gold market, and real estate market. However, each of these sub-markets still has a substantial number of other sub-markets. The classification and selection of financial markets in this study are based on the intricate relaTtionships among sub-markets, data availability, and thorough analytical correctness. The samples used in this study were taken throughout a period of time that ran from 4 January 2016 to 12 November 2021.

#### 3.1.1. Data Description

By adding a first-order difference of natural logarithms to the market’s daily closing price, the formula for calculating the rate of return inside each market is as follows: (the rate of return for sector I at stage t, the closing price for sector I at stage t). Table 2 presents the outcomes.

#### 3.1.2. DCC-GARCH Pre-Modeling Test

##### ADF Test

Table 3 shows that all trades are positive in the average column, but the values are all close to zero, as can be seen from the presentation of the fundamental statistical data in the Table 3. The non-silver finance and light industries are positive, whereas the other industries are negative, according to the skewness column. This implies that while negative shocks are more frequent in some industries, positive shocks are more frequent in non-banking finance and light industry. The Kurtosis column demonstrates that all industries have kurtosis greater than the normal distribution of 3, with the highest peaks in the textile and apparel and defense sectors, demonstrating that the frequency of extreme returns in these two sectors is significantly higher than that of the other industries. Prior to modeling, we evaluate the yield series using the ADF test method to see whether time series are stationary. At a 1% level of significance, the results demonstrate that all variables are stable time series without unit roots.

##### Arch Test

The Ar (1) model is first established for the log-return series in this work, and then the residual ARCH effect of the model is tested. The test findings are displayed in Table 4 with *p* value of 0, rejecting the first hypothesis that there is a strong ARCH effect and allowing the establishment of GARCH model (“*” means that at the significance level of 0.05, if it is larger than the standard value, the original hypothesis is rejected.).

### 3.2. Analysis of DCC-GARCH Model

The dynamic correlation among the Chinese financial submarkets in the two time periods of 2016–2019 and 2019–2021 was determined using the DCC–GARCH model, and the average value of the dynamic correlation throughout the two time periods was calculated to arrive at the conclusion.

#### 3.2.1. Dynamic Correlation Analysis among Financial Sub-Markets in the First Stage

**Figure 1:**
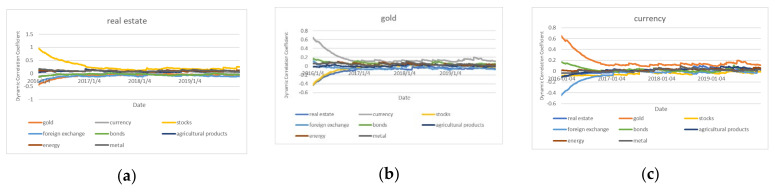

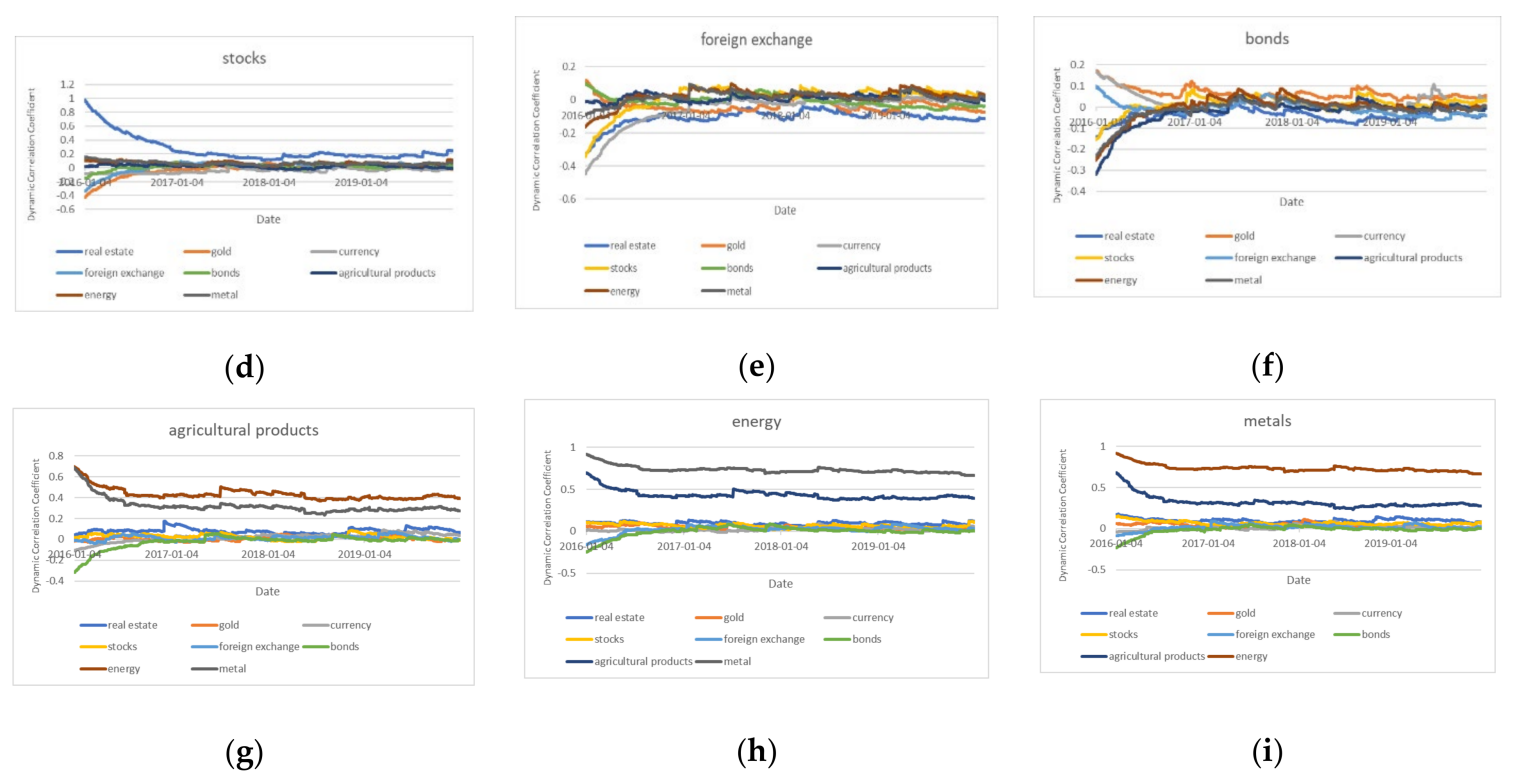
The dynamic correlation test in first stage.

For each market, we ran dynamic correlation tests in the first stage, and the outcomes are displayed in Figure 1. The dynamic correlation test in first stage, (a) means the dynamic coefficients between real estate market and other markets. (b) means the dynamic coefficients between gold market and other markets. (c) means the dynamic coefficients between gold market and other markets. The same as the (d)–(i). Please see date in Table 5.

During the period from 2016 to 2019, there is a large dynamic correlation between the real estate market, the stock market and foreign exchange market, the gold market and currency market, the agriculture market, and the markets for energy and metals. The real estate and stock markets in China have a clear positive dynamic link. Both the stock market and the real estate market are significant elements of China’s economic system from a macroeconomic perspective. The real estate and stock markets in China have a clear positive dynamic link. Both the stock market and the real estate market are significant elements of China’s economic system from a macroeconomic perspective. Economic development will have an impact on the operation and growth of these two markets; from the standpoint of investors, who are heavily involved in these two markets, the movement and allocation of capital between them, as well as their plans and attitudes, will also have an impact. Therefore, it is easy to understand that the Chinese real estate market and the stock market in 2016–2019 are closely related.

As we all know, the “New Normal” stage refers to the pursuit of medium-to-high speed development rather than the high-speed development that was previously pursued in order to accomplish a rebalancing of the economic structure. For example, there are some basic characteristics: (1) Consumption patterns have changed, which indicates that diverse consumption is frequently observed in public life. (2) New technology is continuing to develop as a result of the infrastructure’s ongoing improvements. (3) High-end, mass production is replacing low-cost manufacturing. (4) Emerging industries include media and communications. The dynamic correlation diagram between several markets during the “New Normal” stage of China’s economy reveals that there are significant oscillations at the start of this period. Analyzing the historical context makes it clear that the 2015 stock market meltdown is what precipitated this. In addition, the outbreak continues to have a significant effect on Chinese financial submarkets.

#### 3.2.2. Dynamic Correlation Analysis between Financial Sub-Markets in the Second Stage

**Figure 2:**
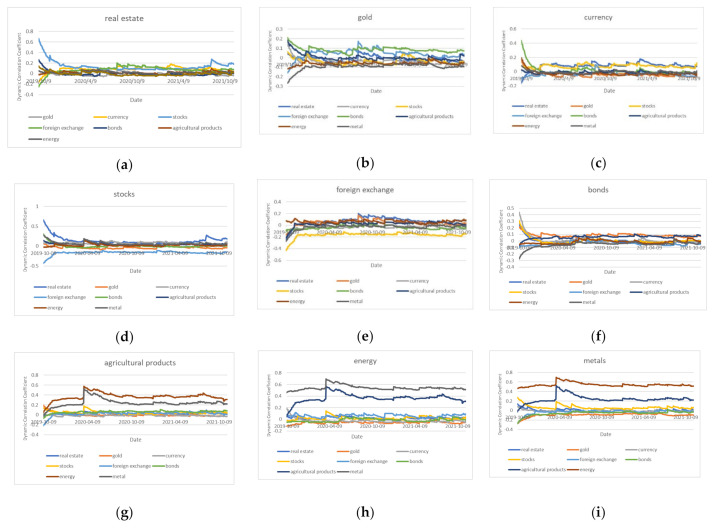
The dynamic correlation test in second stage.

We also conducted dynamic correlation test in the second stage, and the results are shown in Figure 2. The dynamic correlation test in second stage, (a) means the dynamic coefficients between real estate market and other markets. (b) means the dynamic coefficients between gold market and other markets. (c) means the dynamic coefficients between gold market and other markets. The same as the (d)-(i). Please see date in Table 6.

Although there is still a strong positive link between the real estate market and the stock market, the dynamic correlation coefficient among the various financial sub-markets in the stage of 2019–2021 is trending downward in comparison to the stage before it. As China adheres to the policy of “housing without speculation,” the dynamic correlation coefficient for the real estate market has dramatically decreased recently. The government’s macro-control has successfully relieved the contagion of risk factors in the real estate market.

### 3.3. Complex Network Analysis

The dynamic correlation coefficients among China’s financial sub-markets, as determined by the analysis of the DCC-GARCH model, are used to define the nodes and coefficients as complex network points and edges, resulting in a two-stage complex network diagram of China’s financial sub-markets. The topological parameters and minimum span trees of the complex network path contagion analysis can be used to determine the financial risk contagion among various markets.

#### 3.3.1. Data Processing

When building the complex network, the dynamic correlation coefficient between two nodes is set at 5% through calculation and observation of the data in the table. Accordingly, the mean dynamic correlation coefficient below 5% is defined as 0, and the dynamic correlation coefficient above 5% is defined as 1. For the selection of threshold, such as the selection threshold of 1%, through simple calculation, we find that the number of edges is too small, if the threshold selection is too large, the number of edges is too large, these are not suitable for further study. Although the paper of Zhang (2020) is based on Pearson’s coefficient [23], their discussion has some reference for this paper. Of course, the selection of the optimal threshold value will be our next research topic. In Table 7 and Table 8, the values of zero indicate that there is no edge between any two nodes, the values of one indicate there is an edge between any two nodes. This yields Table 7 and Table 8, which represent the zero-one matrix in the two stages. The current literature is used to choose the thresholds. The authors have come to several important conclusions concerning the financial market risk research based on the criteria.

#### 3.3.2. Two-Stage Complex Network Diagram

A network diagram of the two-stage Chinese financial sub-markets is produced by modeling the zero-one matrix.

**Figure 3:**
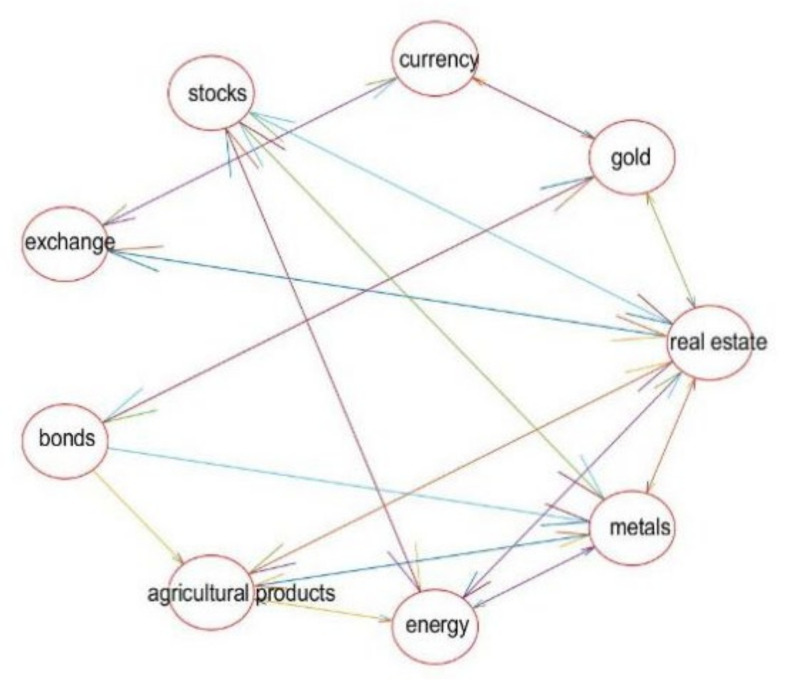
Stage 1: Complex Network Diagram of Financial Markets.

In Figure 3, the real estate market is the most closely tied to other markets in the first stage of the complex network, followed by the metal market, agricultural product market, stock market, and market for agricultural products. It is widely known that China’s real estate market has been at a high level for many years and is a source of concern for investors because the real estate market played a significant part in China’s financial market from 2016 to 2019. As a result, the other markets’ volatility will be impacted by the real estate market’s volatility.

**Figure 4:**
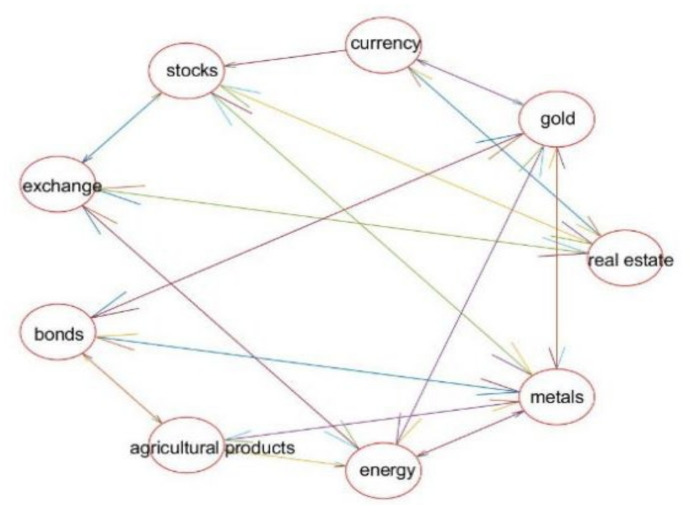
Stage 2: Complex Network Diagram of Financial Markets.

In Figure 4, with the exception of the real estate market, which is still in a close relationship with other markets, the metal market is closely related to other markets in the second stage of the complex network. The real estate sector in China has been steadily expanding as a result of recent stable house prices. Due to the international metal shortage, situation, and fact that metal is a non-renewable resource, the metal market is more volatile under these influences, which also increases the significance of metal’s position in the network.

Through the comparative analysis of the complex network diagrams of the above two-stage financial markets, it can be seen that: (1) First, as a whole, the connection among financial sub-markets and the transmission of volatility risks is closer. (2) According to the principle of “no speculation”, the dynamic correlation between the real estate market and other sub-markets in the pre-epidemic period was closer than that after the COVID-19. (3) Finally, the metal market was closely linked with various financial sub-markets before and after the COVID-19.

#### 3.3.3. Two-Stage Complex Network Topology Parameter Analysis

Table 9 lists the findings from an investigation of the two-stage complex network’s topological parameters. 

Table 9 shows that the network’s average clustering coefficient is higher in the first stage, indicating that local connections are stronger and risks are more likely to spread locally. New connections among nodes or damage to important nodes may be to blame for the network’s increased average path length. The network graph’s size and number of connections among nodes grow as the width of the network graph increases. It demonstrates that the complex network graph is more closely connected in the first stage, while the average path becomes shorter and has a greater diameter in the second stage, demonstrating that the complex network graph grows but the connection among nodes does not. Chinese finance sub-markets in these two stages have strong clustering coefficients and short average path lengths.

#### 3.3.4. Two-Stage Minimum Spanning Tree Diagram Analysis

**Figure 5:**
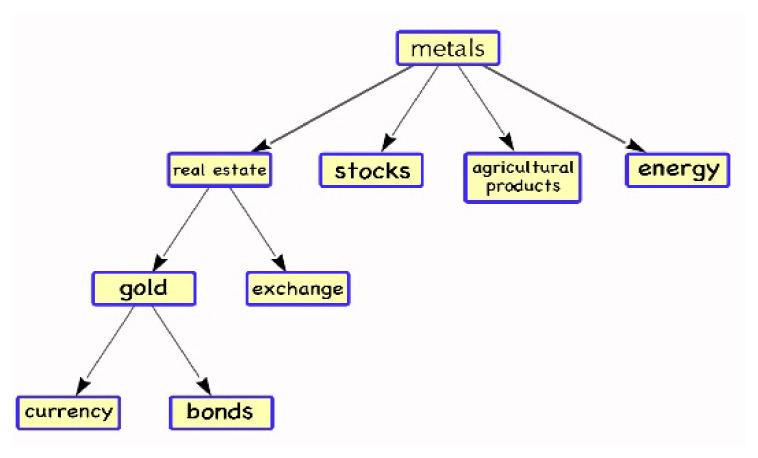
Stage 1: Minimum Spanning Tree Diagram.

Real estate and the foreign exchange market always exhibit a link, as shown in Figure 5. Risk changes in the real estate industry are currently affecting the foreign exchange market. The real estate market and other markets, including the gold market and the foreign exchange market, had a direct association prior to the COVID-19 outbreak. However, it also has an indirect impact on the bond and currency markets. The Chinese philosophy of “housing, not speculating” in recent years, which has restrained the overheating state of the real estate market is related to this problem. In the past, economic growth in the real estate sector was typically driven by investment, with heavy investment concentrating on manufacturing, real estate, and infrastructure investments. Institutions in this sector have a number of heavy industries, including steel, cement, coal, oil and petrochemicals, and building materials, have been fueled by this. As a result, the real estate market, which occupies the connecting link position, has a strong association with the metal, gold, and foreign exchange markets in the first stage.

**Figure 6:**
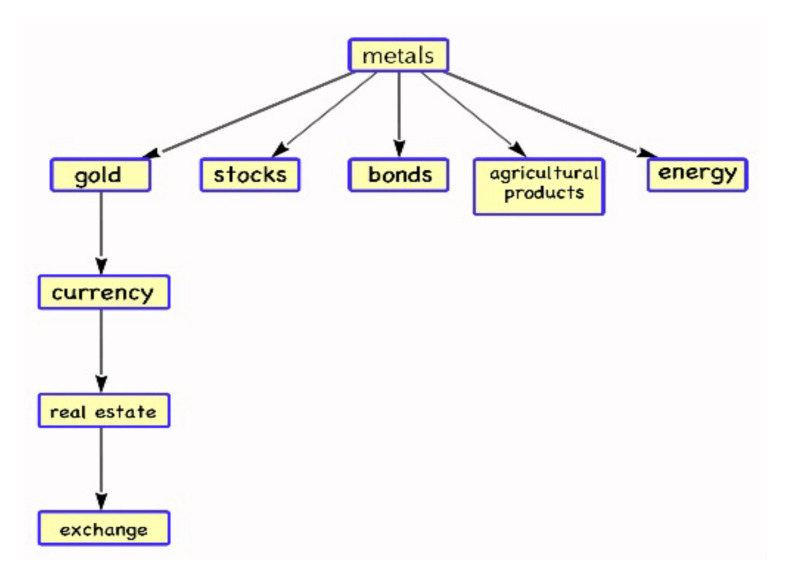
Stage 2: Minimum Spanning Tree Diagram.

Figure 6 shows that although the COVID-19 outbreak has significantly impacted people’s quality of life, from the perspective of financial market fundamentals, it has not yet had a significant negative impact on the Chinese economic growth. However, from the perspective of risk contagion, the risk spillover effect of each sub-market has significantly decreased, and the extreme risk has spread among the sub-markets. Following the COVID-19 outbreak, the risk of contagion among China’s financial sub-markets has generally demonstrated a clear dispersion pattern. The macro policies of China’s financial regulatory authorities have played a significant certain role in mitigating the risks in the face of the extraordinary risk impact of the black swan occurrence.

By comparing the two minimal spanning tree diagrams, it can be shown that from 2016 to 2021, although the COVID-19 pandemic had a significant impact on people’s lifestyles, for the time being, the fundamentals of the financial sector remained stable. Extreme risks have diffused among sub-markets, but from the perspective of risk contagion, the risk spillover effect of each sub-market has not greatly grown. The metal market for bulk commodities has served as the sole primary root point for the yield network of Chinese financial sub-markets throughout this time. The metal, energy, and agricultural product markets are all part of the commodity market. According to the empirical findings, the metal market usually occupies the primary root position in the financial sub-market, demonstrating that it is crucial to the process of risk contagion, which is connected to two things. The following two things define this: (1) The first is that the commodity market is inherently speculative. Futures on commodities immediately benefit the actual economy. The commodity market is highly speculative because investors can utilize leverage to purchase short and sell short. (2) Next, the industrialization process. In China’s economic structure, the secondary industry has long been the cornerstone of encouraging economic progress. One of the main problems facing China’s economy is how to generate high-quality and sustainable growth. The metal market and industry have a close relationship that has helped provide the groundwork for economic progress. And the government is working to change the old high investment growth mode in order to change the economic structure and implement the “housing, not speculating” policy.

The comparison of the empirical results of the two stages reveals that the connection between the real estate market and the metal market has weakened, as has the level of risk contagion, which is no longer at the center. The currency market and interest rate liberalization are at the heart of financial reform, and they have always been inextricably linked to the gold market. Price guidance is weak in the stock market, market manipulation is common, and China’s stock market has policy characteristics. Because of its strong performance, whether in stock market operation or risk transmission, the government has been actively guiding, so that the stock market does not hold a pivotal position in risk transmission in both stages. The bond market is inextricably linked to the currency market, and the development of the Chinese bond market trails that of the stock market.

## 4. Discussion

Our paper is empirical studies focusing on specific datasets, different from the evolution of extreme risk spillovers in China’s financial sub-market by using directed weighted complex networks during the financial crisis [5], We are interested in learning how the Chinese financial sector is handling the COVID-19 outbreak.

Moreover, the dynamic correlation coefficient between markets is mostly discussed using data from two markets through the GARCH-BEKK model [3,9]. We expanded on the discussion of dynamic coefficients by employing the DCC-GARCH model in the complex network, broadening the research field of vision.

Although the models used in this paper are relatively basic in the study of risk contagion, by combining the whole and the individual, we can gain a more comprehensive understanding of the financial markets after the epidemic.

## 5. Conclusions

### 5.1. Empirical Conclusions

The net impact of volatility spillovers in financial submarkets such as the real estate and gold markets has shifted. The changes in the transmission paths of volatility risks in the two financial submarkets to other submarkets suggest that volatility risks in the real estate and metal markets did not cause serious systemic risk contagion. At the same time, risk transmission among Chinese financial markets is closer than it was before COVID-19, but the overall risk transmission path has not changed significantly. The metal market is always in the root position of risk contagion and the net impact state of risk contagion fluctuation overflow.

According to the study’s findings, China’s financial market continues to exhibit the traits of a policy market, which indicates that the financial market is somewhat influenced by the active direction of the government. The position of the real estate market in China’s financial market has significantly altered, and the metal market is its core position, according to a comparison of the risk transmission channels between the two stages of the financial submarket. The findings of this paper are in line with the current state of economic development in China. The Chinese stock market and currency markets should occupy a significant position in the Chinese financial markets, just as the empirical findings suggested. However, they did not have a significant effect on the financial market during the period of the new normal and following the COVID-19. This illustrates how China’s financial reform is progressing because the market is still growing. The government actively influences interest rates and currency rates by various excellent policies, fairly directs capital allocation, and has diverse policy effects on cross-border capital flows and exchange rates, even though the market system is hampered in some ways. These characteristics of China’s financial market have a positive impact on the stability of China’s economy and the rapid recovery of the world economy.

### 5.2. Recommendations

1. Through the research on the normalization stage of the current epidemic, the macro-control of the real estate market has played a certain positive role in the face of such black swan events in China, and it also shows that the risk transmission effect among financial sub-markets is constantly changing. For industries that are in a state of net shock from volatility spillovers, they should continue to strengthen their supervision and regulatory control.

2. Regarding the unique position of the metal market, authorities must pay particular attention to its risk status as well as the domestic and international metal markets, which are tied to the metal market. At this time, the metal market is an industry that is experiencing net shock from swings. Both the internal risk management of financial institutions and the commodity market should be closely monitored concurrently. And the metal market, which is experiencing an excess of volatility on a net basis, indicating that external concerns should also be taken into consideration.

3. The internal operating characteristics of various markets must be taken into consideration for the presence of their own links among them, and comprehensive financial market oversight must be carried out.

4. After the COVID-19 outbreak, China’s dynamic zeroing policy and the characteristics of the financial market policy, so that China’s financial market is still in a relatively stable stage, were important considerations for policymakers in China and throughout the world. Despite the impact of the epidemic, the current development trend of the domestic capital market is still strong. The real estate and infrastructure have always been an important position, so global investors can give some attention.

## Figures and Tables

**Table 1:** Financial sub-markets classification and index selection.

Primary Market	Secondary Market	Specific Index
capital market	stock market	CSI 300 Index
	bond market	Chinese bond index
money market	interbank market	Inter-bank overnight rate
Foreign exchange market	US dollar to RMB	US dollar to RMB exchange rate
commodity market	metal market	south China Metal Index
	energy market	south China Energy Index
	agricultural products market	agricultural products index
gold market		AU9995 gold spot price
real estate market		Shanghai Real Estate Sector Index

**Table 2:** Financial sub-markets log-return descriptive statistics.

Market	Mean	Standard Deviation	Skewness	Kurtosis	Jarque-Bera	*p*
stocks	0.000	0.016	−0.147	7.681	1120.128	0.000
bonds	0.000	0.012	−0.542	10.200	2699.559	0.000
currency	0.000	0.009	0.034	8.063	1305.579	0.000
exchange	0.000	0.119	1.030	13.419	5743.428	0.000
metals	0.001	0.014	−0.319	6.963	820.360	0.000
energy	0.001	0.002	−0.098	5.109	228.446	0.000
agricultural products	0.000	0.014	−0.218	6.662	692.681	0.000
gold	0.000	0.008	1.590	25.943	27,317.020	0.000
real estate	0.000	0.001	0.781	17.752	11,204.740	0.000

**Table 3:** ADF Test results (“*” represents the result at a significant level of 0.05).

Market	ADF	Prob. *
stocks	−35.736	0.000
bonds	−25.418	0.000
currency	−25.401	0.000
exchange	−33.304	0.000
metals	−34.580	0.000
energy	−34.790	0.000
agricultural products	−36.773	0.000
gold	−34.404	0.000

**Table 4:** Arch test results.

**F-Statistic**	24.344	**Prob.F (1,1219)**	0.000
Obs * R-Squared	23.907	**Prob.Chi-Square (1)**	0.000

**Table 5:** The dynamic correlation data in first stage.

	Real Estate	Gold	Currency	Stocks	Foreign Exchange	Bonds	Agricultural Products	Energy	Metals
**real estate**	1.000	−0.090	0.010	0.260	−0.110	−0.040	0.080	0.090	0.100
**gold**	−0.090	1.000	0.170	−0.030	−0.040	0.070	0.010	0.040	0.040
**currency**	0.010	0.170	1.000	−0.040	−0.050	0.030	0.020	0.020	0.010
**stocks**	0.260	−0.030	−0.040	1.000	0.010	0.010	0.020	0.070	0.060
**foreign exchange**	−0.110	−0.040	−0.050	0.010	1.000	−0.010	0.010	0.020	0.020
**bonds**	−0.040	0.070	0.030	0.010	−0.010	1.000	−0.020	0.000	−0.010
**agricultural products**	0.080	0.010	0.020	0.020	0.010	−0.020	1.000	0.430	0.320
**energy**	0.090	0.040	0.020	0.070	0.020	0.000	0.430	1.000	0.730
**metals**	0.100	0.040	0.010	0.060	0.020	−0.010	0.320	0.730	1.000

**Table 6:** The dynamic correlation data in second stage.

	Real Estate	Gold	Currency	Stocks	Exchange	Bonds	Agricultural Products	Energy	Metals
**real estate**	1.000	−0.010	0.080	0.150	0.070	0.000	0.030	0.020	0.000
**gold**	−0.010	1.000	−0.050	−0.030	0.040	0.090	0.010	−0.060	−0.100
**currency**	0.080	−0.050	1.000	0.080	−0.030	0.020	−0.010	−0.020	−0.010
**stocks**	0.150	−0.030	0.080	1.000	−0.170	0.010	0.040	0.020	0.070
**exchange**	0.070	0.040	−0.030	−0.170	1.000	−0.040	0.020	0.060	−0.030
**bonds**	0.000	0.090	0.020	0.010	−0.040	1.000	0.060	−0.010	−0.060
**agricultural products**	0.030	0.010	−0.010	0.040	0.020	0.060	1.000	0.360	0.240
**energy**	0.020	−0.060	−0.020	0.020	0.060	−0.010	0.360	1.000	0.540
**metal**	0.000	−0.100	−0.010	0.070	−0.030	−0.060	0.240	0.540	1.000

**Table 7:** The first stage of the zero-one matrix.

	Real Estate	Gold	Currency	Stocks	Exchange	Bonds	Agricultural Products	Energy	Metals
**real estate**	0	1	0	1	1	0	1	1	1
**gold**	1	0	1	0	0	1	0	0	0
**currency**	0	1	0	0	1	0	0	0	0
**stocks**	1	0	0	0	0	0	0	1	1
**exchange**	1	0	1	0	0	0	0	0	0
**bonds**	0	1	0	0	0	0	1	0	1
**agricultural products**	1	0	0	0	0	0	0	1	1
**energy**	1	0	0	1	0	0	1	0	1
**metals**	1	0	0	1	0	0	1	1	0

**Table 8:** The second stage of the zero-one matrix.

	Real Estate	Gold	Currency	Stocks	Exchange	Bonds	Agricultural Products	Energy	Metals
**real estate**	0	0	1	1	1	0	0	0	0
**gold**	0	0	1	0	0	1	0	1	1
**currency**	1	1	0	1	0	0	0	0	0
**stocks**	1	0	1	0	1	0	0	0	1
**exchange**	1	0	0	1	0	0	0	1	0
**bonds**	0	1	0	0	0	0	1	0	1
**agricultural products**	0	0	0	0	0	1	0	1	1
**energy**	0	1	0	0	1	0	1	0	1
**metals**	0	1	0	1	0	1	1	1	0

**Table 9:** The results of the topology parameters.

	Average Clustering Coefficient	Average Path Length	Diameter
**Stage 1**	0.444	1.861	3.111
**Stage 2**	0.350	1.694	3.444

## Data Availability

Not applicable.
